# MOGAD Involving Cranial Neuropathies: A Case Report and Review of Literature

**DOI:** 10.3390/brainsci12111529

**Published:** 2022-11-11

**Authors:** Yangsa Du, Ling Xiao, Zijin Ding, Kailing Huang, Bo Xiao, Li Feng

**Affiliations:** 1 Department of Neurology, Xiangya Hospital, Central South University, Changsha 410008, China; 2 Department of Anesthesiology, Xiangya Hospital, Central South University, Changsha 410008, China; 3 Department of Neurology, Xiangya Hospital, Central South University (Jiangxi Branch), Nanchang 330000, China; 4 National Clinical Research Center for Geriatric Diseases, Xiangya Hospital, Central South University, Changsha 410008, China

**Keywords:** myelin oligodendrocyte glycoprotein, MOGAD, anti-MOG, cranial neuropathies, optic neuritis

## Abstract

Myelin-oligodendrocyte glycoprotein (MOG) antibody-associated disease (MOGAD) is an autoimmune-mediated demyelinating disease of the central nervous system (CNS). Patients with MOGAD may develop any combination of optic neuritis (ON), myelitis, brainstem syndrome and encephalitis. Reports of MOGAD with cranial nerve involvement are rare. Herein, we report a MOGAD patient with cranial neuropathies. In addition, we summarized the clinical features of the previously reported six MOG-IgG-positive cases with cranial nerve involvement and discussed the underlying mechanisms of MOGAD involving cranial nerves. Cranial neuropathy is an emerging phenotype in MOGAD, which has characteristics of both central and peripheral nervous system (PNS) involvement, with the trigeminal nerve being the most commonly affected nerve. MOG antibody testing in patients with cranial neuropathies is warranted, and immunotherapy is advocated when the risk of relapse is high. Although higher antibody titers and persistently positive serological test results are predictive of disease recurrence, the long-term outcomes of MOG-IgG-positive patients with cranial neuropathies remain largely unknown.

## 1. Introduction

MOGAD, an inflammatory demyelinating disorder of the central nervous system mediated by antibodies, has been recognized as a novel inflammatory disease entity independent of neuromyelitis optica spectrum disorders (NMOSD) and multiple sclerosis (MS) over the last few years [1], which is estimated to have an annual incidence of 1.6/million person years (3.1/million in children; 1.3/million in adults), similar to that of aquaporin-4 (AQP4)-IgG+NMOSD [2].

MOG, a molecule found on the external membrane of myelin sheaths, is demonstrated to regulate the stability of oligodendrocyte microtubule, maintain the structural integrity of the myelin sheath, and more importantly, function as an essential target of autoantibodies and cell-mediated immune responses in MOGAD [3]. It is believed to exist mainly within the CNS and the optic nerves. As a result, the clinical phenotype of MOGAD includes ON, myelitis and brainstem syndrome, and sometimes encephalitis and fulminant disease progression [4]. However, there have been rare reports of cranial nerve (CN) involvement in MOGAD [5,6,7,8]. This uncommon phenomenon has been proposed but not fully described and researched, with only one preliminary immunohistochemistry study on macaque tissue conducted to explore the underlying mechanism of CN involvement [7].

Here we described the clinical and laboratory features of a MOGAD patient with cranial neuropathies. Furthermore, we reviewed the literature on this clinical phenotype, summarized the clinical features, and explored the underlying mechanisms of CN involvement in MOGAD. Our work comprehensively portrayed the new and expanded MOGAD phenotype, thereby helping to better investigate, diagnose and treat this disease.

## 2. Case Presentation

**Figure 1:**
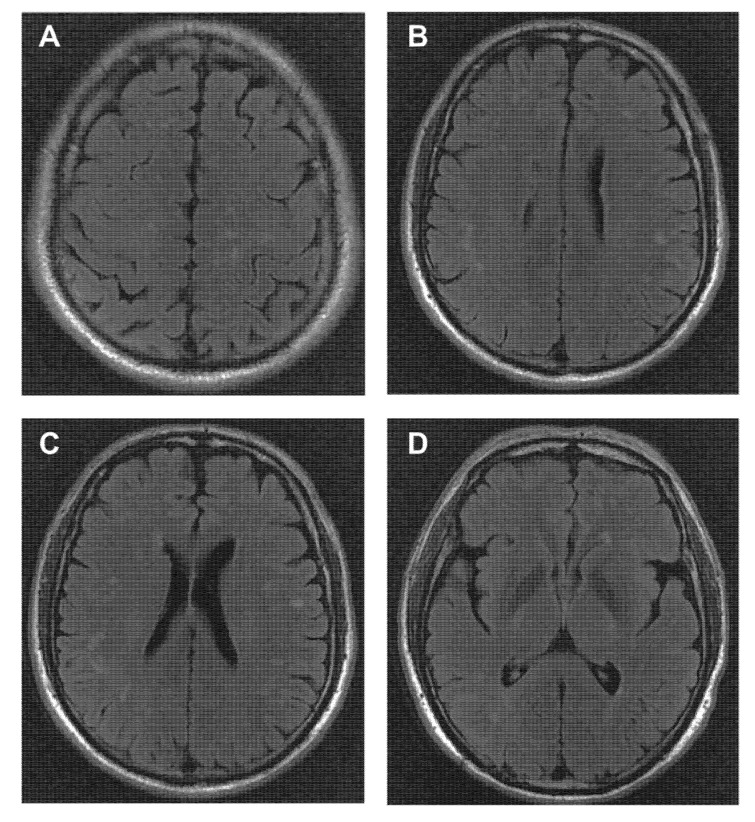
Radiologic features of MOG-IgG-positive patients with cranial neuropathies. T2-FLAIR hyperintensities in the subcortical white matter of bilateral frontal lobe (**A**); bilateral frontal and parietal lobe (**B**,**C**); and right occipital lobe near the posterior horn of the lateral ventricle (**D**).

A 64-year-old man was admitted to our department for decreased bilateral vision, ocular pain, and weak right eye closure for 13 days. The patient’s weight was 65 kg and he denied significant changes in weight and appetite. He had a past medical history of gastric ulcers and was a habitual smoker, had smoked five cigarettes per day for 20 years, and quit smoking for five years. Ophthalmological examination revealed that visual acuity was 0.3 in the right eye and 0.4 in the left. Overt lens opacities were present in the bilateral eyes, and his visual field, pupillary reactions, and the appearance of the optic nerve head were normal. Neurologic examination revealed restricted inward movement of the left eyeball, right blepharoptosis, right facial hypesthesia, and weak tongue movement. Electromyography (EMG) of the right peripheral facial nerve showed low amplitude with the amplitudes for the temporal, zygomatic, buccal, and marginal mandibular branches of the facial nerve declining below 10% than the contralateral side, suggesting neurogenic damages in the right facial muscles. Visual-evoked potential showed prolonged P100 latency in both eyes, and brainstem auditory-evoked potential suggested affected left auditory pathways. Brain magnetic resonance imaging (MRI) identified multiple hyperintense T2-weighted and T2- fluid-attenuated inversion recovery (FLAIR) lesions near the anterior and posterior horn of the bilateral lateral ventricles, and in subcortical area of bilateral frontal, temporal, parietal, and occipital lobes (Figure 1). The lesions were not enhanced. The Cerebrospinal fluid (CSF) protein was within the normal range (0.27 g/L) without pleocytosis or oligoclonal bands (OCBs). Screening for serum and CSF antiganglioside and paraneoplastic antibodies was all negative. The routine blood test and erythrocyte sedimentation rate were normal. The T-SPOT.TB assay for tuberculosis, multiple virus antibodies, the urea breath test (UBT) for H. pylori infection were negative. Toxic-metabolic diseases, diabetes mellitus, and vitamin deficiencies were excluded by laboratory investigation. Elevated serum MOG-IgG antibodies (titer 1:100) were detected using a clinically validated microscopic live cell-based assay [2]. In consideration of diminished vision and abnormal visual- and auditory-evoked potential, the involvement of brainstem could not be ruled out, even without any imaging structural abnormalities. In addition, the patient experienced limited ocular motility, facial numbness, dysarthria, and dysphagia, as well as right facial nerve damage according to EMG testing, and we proposed the diagnosis of possible MOGAD with multiple cranial neuropathies. The patient refused further imaging of cranial nerve roots for economic reasons. Methylprednisolone with a dose of 500 mg/day for three days was administered and followed by oral prednisone and mycophenolate mofetil. At the six-month follow-up, the patient gradually recovered, and his visual acuity was improved to 0.5 at the right eye and 0.55 at the left eye, with left slightly right facial hypesthesia.

## 3. Discussion

MOGAD is a recently recognized demyelinating autoimmune disorder predominantly affecting the optic nerve and spinal cord, and brainstem involvement is common in patients with MOG-IgG-related ON and/or myelitis [9]. More recently, new clinical phenotypes such as cranial nerve involvement have been reported, and the underlying pathophysiology is not clear [7,8]. MOG is considered to be a protein expressed by oligodendrocytes that only exist in the central nervous system (CNS), thus its presence in peripheral tissue is not expected [3]. However, MOG-IgG-positive patients with radiologic and/or clinical cranial nerve involvement are increasingly identified. To gain a better understanding of this rare phenotype, we not only reviewed six other cases in the literature to investigate the characteristics of clinical presentation, radiologic features, treatment and prognosis of MOG-IgG-positive patients with cranial neuropathies (Table 1), but also summarized the possible underlying pathophysiology.

### 3.1. Clinical Presentation

Cranial neuropathies of any etiology commonly presented with the corresponding symptoms of cranial nerve deficits. In MOGAD patients, the trigeminal nerve was the most frequently involved cranial nerve (five patients), which is mainly manifested as facial sensory disturbances without laterality. Oculomotor nerve impairment was also in half of the patients, with ocular movement limitation and diplopia. Kawakami et al. presented a pediatric MOG-IgG-positive patient who developed acute onset of left ptosis and was initially diagnosed with isolated oculomotor nerve impairment [11]. Because of the lack of obvious lesions on imaging, this case suggests primary peripheral myelin demyelination instead of a process secondary to CNS inflammation. Other less frequent cranial neuropathies include trochlear (1/6) and abducent nerve (1/6) impairment presented with restricted ocular movement, facial nerve impairment (1/6) presented with inadequate eyelid closure and vestibulocochlear nerve impairment (1/6) presented with vertigo. Besides, our patient had blepharoptosis, inadequate eyelid closure, facial hypesthesia and tongue motor impairment, suggesting involvement of oculomotor, facial, trigeminal, and hypoglossal nerves.

In terms of manifestations related to CNS lesions in these patients, one patient with cervical spine, thoracic spine, and conus medullaris involvement presented with numbness and weakness of limbs, loss of bladder and bowel control, and erectile dysfunction. One patient showing meningeal involvement had fever, recurrent headaches, and seizures. Imaging showing intracranial white matter demyelination may be accompanied by gait instability and cognitive impairment.

MOGAD is reportedly more prevalent in adolescents and young adults [2]. In comparison, the onset age of MOG-IgG-positive patients with cranial neuropathies ranged from 51 to 76 years old in the majority (five patients). More cases are need to confirm this disparity.

### 3.2. Disease Course

MOGAD has been generally considered to have a monophasic disease course. Between 30–80% of patients, however, may experience relapse after disease onset [12]. Male patients with myelitis experienced a lower relapse risk. Other predictors include prolonged steroid treatment and the MOG-antibody negative seroconversion [13]. Among the seven MOG-IgG–positive patients with cranial neuropathies in this review, two had a relapsing disease course both with ON, and the other five remained monophasic. Our results are consistent with the view that the onset phenotype of ON may have a tendency for an elevated risk of relapse [14]. The child with isolated oculomotor nerve impairment was also monophasic [11].

### 3.3. CSF Examination

CSF in serum MOG-IgG-positive patients shows normal to mildly elevated intracranial pressure, normal to slightly elevated protein levels and lymphocytic pleocytosis, as well as rare OCBs. In this review, two patients had CSF pleocytosis, four increased protein levels, and one OCBs. CSF analysis of the MOG-IgG-positive girl with isolated oculomotor neuritis without CNS involvement revealed normal cells, protein levels and OCBs [11].

### 3.4. MRI Features

Neuroimaging findings of MOGAD with cranial neuropathy vary depending on the lesion site. Lesions were commonly located in the cortical matter, deep and subcortical white matter, anterior optic nerve, the conus and central grey matter of spinal cord [15]. Affected cranial nerves were enhanced presumably because of the inflammatory response and increased vascular permeability during the acute phase. There was also one patient with normal neuroimaging, indicating possible subclinical alterations [10]. Although some studies suggest that 18-fluorodeoxyglucose positron emission tomography (FDG-PET) can play a potential role in diagnosis of MOGAD [6,16], FDG-PET imaging in two patients showed no abnormalities [7]. Because of its low sensitivity in intracranial tiny or diffuse lesions, PET is not recommended as a routine examination.

### 3.5. Treatment and Outcome

To date, the treatment of MOGAD with cranial neuropathy is no different than that for MOGAD without nerve involvement. High-dose glucocorticoid is the routine regime during the acute phase followed by a tapering course of oral prednisolone. MOGAD is hormone-dependent, so relapses tend to occur early, often soon after corticosteroids are stopped. When the hormone reduction time is less than three months, the possibility of relapse doubles [14,17]. Two of the seven patients in this review relapsed, one within three months and one after hormone treatment. Therefore, it is recommended to prolong the reduction of corticosteroids and maintain low-dose steroid therapy for at least three months. In case of the severe initial episode with a poor prognosis, a shift toward prolonged immunotherapy can be considered. AZA, MMF, and rituximab were recommended in multiple studies. Regular intravenous immunoglobulin G (IVIG) and tocilizumab might be beneficial [18]. The treatment regimens should be individualized, taking into account the characteristics of patients, clinical phenotype, and drug safety.

The majority of patients with MOGAD can entirely or almost entirely recover, which is similar to MS. Despite that some MOGAD patients with severe optic nerve damage undertook irreversible visual deficits, the long-term visual outcomes were much more desirable than those of AQP4-IgG+NMOSD patients [19]. During the follow-up, four patients recovered, and the symptoms completely disappeared, one patient had mild residual neurogenic bladder and erectile dysfunction at three months, and two patients had mild facial numbness. Two patients developed new-onset lesions on a routine brain MRI but had no clinical symptoms. For MOG-IgG-positive patients with cranial or peripheral nerve involvement, the combination of hormone drugs and immunosuppressive therapy is proven to be effective, which could improve clinical symptoms and reduce relapses.

### 3.6. Possible Underlying Pathophysiology

The pathophysiology of MOGAD is summarized below. Unknown triggers first stimulate the production of anti-MOG antibodies, anti-MOG antibody-producing cells and antigen-specific T follicular helper cells, which then cross the blood-brain barrier and bound to MOG on myelin, resulting in demyelination [4]. Since MOG only exists on the surface of myelin sheaths and oligodendrocyte membranes in the CNS in humans, the mechanism of MOGAD with cranial neuropathies which are lesions in the PNS has raised much attention and prompted further research.

One hypothesis is that MOG as the target of immune response is lowly expressed in the PNS, which is supported by a study detecting MOG at the cytoplasmic in the PNS of rodents and primates [20]. However, the binding epitopes of MOG antibodies in rodents were apparently different from humans [21]. Cobo-Calvo et al. used nonhuman primate tissues to explore MOG-IgG binding in CN and found no labeling, suggesting that it is less probable that MOG antibodies directly drive the process of immune-mediated cranial neuropathies [7]. A possible explanation for CN involvement is that coexisting, and yet unidentified autoantibodies target the cranial nerves. There have been reports of the coexistence of multiple autoantibodies in autoimmune encephalitis and demyelination [22], and in an Australasian MOGAD cohort, serum antibodies targeting neurofascin 155(NF155), contactin-associated protein 2(CASPR2), and Ganglioside GM1(GM1) were detected in MOGAD patients with PNS involvement [21]. Other hypotheses include the inflammatory process secondary to the intra-axial pontine injury, autoimmunity in CN triggered by the secreted isoform of MOG through molecular mimicry with peripheral myelin proteins, or increased susceptibility to autoimmunity in some patients [8], which need more research to confirm.

## 4. Conclusions

Cranial neuropathy, either isolated or accompanied by CNS involvement, is an emerging phenotype in MOGAD, which appears to be associated with older age at disease onset. Currently, there is no uniform standard for the diagnosis of MOG-IgG with cranial nerve involvement. This phenotype has features of both central and peripheral nervous system involvement, with the trigeminal nerve being the most commonly affected nerve. Testing of MOG antibody in patients with cranial neuropathies is necessary, and immunotherapy is advocated when relapse risk is high. Higher antibody levels and persistent positive serologic test results can predict disease recurrence, but the long-term outcomes of MOG-IgG-positive patients with cranial neuropathies remain unclear.

The main limitation of this study is that it was based on a small number of studies, and mainly case reports. Future studies with larger-scale sizes and longer follow-up times are needed to better characterize the epidemiologic and clinical features, treatment and long-term prognosis of this unique phenotype.

## Figures and Tables

**Table 1:** Summary of the case reports that targeted MOG-associated cranial neuropathies.

Patient	Onset Age and Sex	Main Clinical Presentation	Optic Neuritis		Other Cranial Nerve Impairment		Serum MOG-lgG	CSF Examination			Lesion Location in Brain and Spinal Cord MRI	Immuno-SuppressiveTreatment	Disease Course	Article
			+/−	Evidence	Cranial Nerves	Evidence		Cells	Proteins (g/L)	OCBs				
1	76/M	Left facial numbness, dysmetria of the left arm	−	/	Ⅴ (left)	Left trigeminal nerve enhancement at the root level	+	0	0.52	−	Both upper cerebellar peduncles Left trigeminal nerve	None	Monophasic course	[7]
2	16/F	Fever, general mental alteration, disorientation, nystagmus and right visual acuity impairment with retro-orbital pain.	*	/	Ⅲ (bilateral)	Both third oculomotor nerves enhancement	+	0	normal	−	Bilateral juxtacortical and supratentorial white matter, both third oculomotor nerves cervical	None	Relapsing course	[7]
3	52/F	Diplopia, vertigo, and gait instability	−	/	Ⅴ (bilateral) Ⅷ (bilateral)	A bilateral trigeminal and vestibulocochlear nerve enhancement	+	27	0.51	−	The medulla oblongata and pons and the dorsal pontine tegmentum, bilateral trigeminal and vestibulocochlear nerve	None	Monophasic course	[7]
4	51/F	The blurring of vision and diplopia of the right eye, numbness of the right forehead, right visual acuity impairment	+	Optical coherence tomography(OCT )showed thinning of the right temporal fibers and visual evoked potential showed delayed P100 on the left and absent P100 on the right.	Ⅲ, Ⅳ, Ⅴ *, Ⅵ (right)	Hess chart showed under-action of all extra-ocular muscles of the right eye	1:10	/	/	/	MRI brain did not show any lesion in the brainstem or the nerve roots	Azathioprine(AZA)	Relapsing course	[10]
5	51/F	Right facial numbness and weakness	−	/	Ⅴ (bilateral)	Bilateral trigeminal enhancement	1:512	78	0.47	−	Bilateral trigeminal, the dorsal medulla oblongata, both middle cerebellar peduncles and aqueduct of midbrain	None	Monophasic course	[5]
6	18/M	Right facial numbness, numbness and weakness in limbs, loss of bladder and bowel control, and erectile dysfunction	−	/	Ⅴ (right)	Trigeminal nerve enhancement	+	20	0.8	6	Supratentorial and infratentorial brain structures, cervical spine, thoracic spine, and conus medullaris and lumbosacral nerve roots.	None	Monophasic course	[8]
7	64/M	Decreased bilateral vision, restricted inward movement of the left eyeball, right blepharoptosis right facial hypesthesia and weak tongue movement.	+	Visual-evoked potential showed prolonged P100 latency in both eyes	Ⅲ(left) * Ⅴ *,Ⅶ (right), Ⅻ (bilateral) *	EMG of the right peripheral facial nerve showed the amplitudes of the facial nerve declining below 10% than the contralateral side	1:100	0	0.27	−	Bilateral lateral ventricles, bilateral frontal, temporal, parietal and occipital subcortices	Mycophenolate mofetil(MMF)	Monophasic course	Patient 1

* Clinical suspicion. Abbreviations: OCBs = oligoclonal bands; Ⅲ = oculomotor nerves; Ⅳ = trochlear nerve; Ⅴ = trigeminal nerve; Ⅵ = abducens nerve; Ⅶ = facial nerve; Ⅷ = vestibulocochlear nerve; Ⅻ = hypoglossal nerve; + = positive; − = negative; / = not report.

## Data Availability

The data generated or analyzed during this study are included in this article.
